# A cluster randomised trial to evaluate a physical activity intervention among 3-5 year old children attending long day care services: study protocol

**DOI:** 10.1186/1471-2458-10-534

**Published:** 2010-09-08

**Authors:** Meghan Finch, Luke Wolfenden, Philip J Morgan, Megan Freund, Rebecca Wyse, John Wiggers

**Affiliations:** 1 Hunter New England Population Health, Newcastle, NSW, Australia; 2 School of Medicine and Public Health University of Newcastle, Newcastle, NSW, Australia; 3 School of Education, University of Newcastle, Newcastle, NSW, Australia; 4 Hunter Medical Research Institute, Newcastle, NSW, Australia

## Abstract

**Background:**

Young children are not participating in recommended levels of physical activity and exhibit high levels of sedentary behaviour. Childcare services provide access to large numbers of young children for prolonged periods, yet there is limited experimental evidence regarding the effectiveness of physical activity interventions implemented in this setting. The aim of this study is to assess the effectiveness and acceptability of a multi-component physical activity intervention, delivered by childcare service staff, in increasing the physical activity levels of children attending long day care services.

**Methods/Design:**

The study will employ a cluster randomised controlled trial design. Three hundred children aged between 3-5 years from twenty randomly selected long day care services in the Hunter Region of New South Wales, Australia will be invited to participate in the trial. Ten of the 20 long day care services will be randomly allocated to deliver the intervention with the remaining ten services allocated to a wait list control group. The physical activity intervention will consist of a number of strategies including: delivering structured fundamental movement skill activities, increasing physical activity opportunities, increasing staff role modelling, providing children with a physical activity promoting indoor and outdoor environment and limiting children's small screen recreation and sedentary behaviours. Intervention effectiveness will be measured via child physical activity levels during attendance at long day care. The study also seeks to determine the acceptability and extent of implementation of the intervention by services and their staff participating in the study.

**Discussion:**

The trial will address current gaps in the research evidence base and contribute to the design and delivery of future interventions promoting physical activity for young children in long day care settings.

**Trial registration:**

Australian New Zealand Clinical Trials Registry ACTRN12610000087055

## Background

Regular physical activity among young children can contribute to social, psychological and fundamental motor skill development, maintain bone health and prevent obesity [1-6]. Despite these benefits, research suggests that preschool aged children are not adequately physically active [3,7,8]. For example, a recent study found that 44% and 21% of Australian preschool aged children are not sufficiently active on weekdays and weekends respectively [8].

For a variety of reasons, childcare services (centre based care including long day care services and pre-school) have been identified as a promising setting for the delivery of interventions to increase physical activity among children in early childhood [2,9-12]. First, childcare services provide access to a large and growing number of children for prolonged periods each day [5,13,14]. Second, childcare services have existing infrastructure which can be used to facilitate child physical activity [13]. Third, childcare service staff appear amenable to interventions which aim to enhance children's activity [15,16]. Lastly, descriptive research suggests that service policies and practices and the physical environment of childcare services are important influences on children's physical activity behaviours [9-11,17].

Despite the potential of childcare services as a setting to increase child physical activity, experimental research examining the effectiveness of physical activity interventions in this setting is limited [12,18]. A recent systematic review identified just two randomised controlled trials that aimed to increase children's physical activity levels in childcare[12]. The first, conducted by Alhassan and colleagues [19] reported no change in physical activity levels of Latino children attending a single preschool following an intervention to increase outdoor play time by 60 minutes. The second trial [20], however, found increased physical activity levels among children of one preschool attending classes where staff were trained to integrate movement experiences into the daily indoor programs compared to those attending classes where teachers did not receive training. While such trials provide some evidence regarding the efficacy of specific intervention strategies, the effectiveness of comprehensive, service-level interventions, which are consistent with best practice physical activity guidelines in this setting [2,21] have not been tested.

The aim of this study is to assess the effectiveness and acceptability of a multi-component physical activity intervention, delivered by childcare service staff, in increasing the physical activity levels of children attending long day care services. This paper will describe the study protocol by which this trial will be conducted.

## Methods/Design

### Study Design

**Figure 1:**
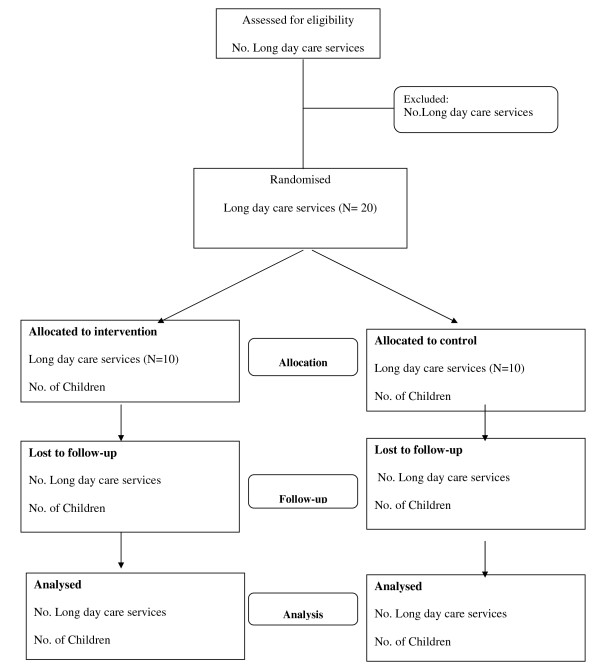
**CONSORT Flowchart describing progress of participants through the trial**.

The study will employ a cluster randomised controlled trial design (see Figure 1). A sample of eligible long day care services in the study region will be randomly selected and approached to participate in the trial. Ten services will be randomly allocated to a service-level physical activity intervention, delivered over a 15 week period, and ten services will be allocated to a wait list control group. The primary trial outcome measure, mean child step counts per minute, will be collected at baseline and approximately 6 months following baseline data collection. Services allocated to the wait list control group will receive the intervention after the collection of all follow-up data.

The research methods will be reported in accordance with the CONSORT statement [22]. The trial is funded by Hunter New England Population Health, and by a Hunter Medical Research Institute Grant (G0900142). Ethical approval to conduct the study has been obtained from the Hunter New England Area Human Research Ethics Committee (approval No.06/07/26/4.04) and University of Newcastle Human Research Ethics Committee (approval No.20100038).

### Setting

The study will take place in the Newcastle, Lake Macquarie and Port Stephens local government areas of the Hunter Region of New South Wales, Australia. These areas encompass non-metropolitan 'major cities' and 'inner regional' areas as described by the Australian Standard Geographic Classification system [23]. There are 385,376 people residing in the area of which 14,061 are children aged 3 to 5 years [24]. Five percent of residents speak languages other than English and two percent of residents are of Aboriginal or Torres Strait Islander origin [24]. The Hunter Region has lower indices of socio-economic status than the New South Wales state average [23].

### Sample

#### Long day care services

Long day care services in NSW provide centre based care for eight or more hours per day for five days per week and usually enroll children from six weeks old to under six years [14]. These services provide specific preschool programs for children aged three to five years that aim to provide early educational activities to help children prepare for school [14].

There are a total of 85 long day care services in the study region. Twenty of these long day care services (24%) will be recruited into the trial. A list of all long day care services in the region provided by the New South Wales Department of Community Services (the Government Licensing Authority) will serve as the sampling frame. Services catering solely for special needs populations, such as children with vision or hearing impairment, will be excluded from participating in the trial given the specialist care required for such children and the likelihood of a differential effect of the intervention in this population group. To be eligible to participate in the trial long day care services will be required to have at least 25 enrolled children aged three to five years. Eligibility will be confirmed with the Authorised Supervisors (managers) of the services during phone contact as part of the recruitment process.

#### Children

Parents of all eligible children aged three to five years at each of the 20 services will be asked to provide consent for child participation in the study. A minimum of 175 children in each of the intervention and control groups at baseline are expected to participate in the study (average of 18 per service) on the basis of consent rates from similar studies in this setting [19]. Children at the service with a significant physical or intellectual disability will be excluded where this disability prohibits or has the potential to preclude participation in the intervention or impair accuracy of physical activity measures. To be eligible, children must be enrolled to attend the service on the day of the week nominated by the Authorised Supervisor for baseline data collection.

### Recruitment Procedures

#### Long day care services

Prior to formal requests to participate, the research trial will be promoted to Authorised Supervisors through existing childcare networks via a postal newsletter, and an email to all long day care services approximately six weeks and two weeks prior to commencing recruitment respectively.

The order in which eligible services in the study region will be approached to participate in the study will be randomised using a random number feature in Microsoft Excel.

Authorised Supervisors will be mailed recruitment letters informing them of the study and requesting their consent to participate. Consent will be obtained through the supervisor faxing or posting a signed consent form back to the research team. If consent is not received within two weeks a research assistant will telephone Authorised Supervisors to answer any questions they may have and remind them to return their form. Recruitment of services will continue until 20 services consent to participate in the study.

#### Children

To maximise child participation in data collection at participating long day care services, the study will employ strategies recommended for obtaining active parental consent for health research within a school setting [25]. Specifically, the recruitment of participants will include the following components:

##### 1. Recruitment oversight

One member of the research team will act as a designated recruitment coordinator and will be the primary liaison with Authorised Supervisors throughout the study. The coordinator will manage the distribution of consent and information materials to services and parents, and monitor return rates of service and parent consent forms. During the recruitment period, parents and Authorised Supervisors will be able to contact the coordinator directly with any queries about the recruitment. The coordinator will not be involved in the delivery of the intervention or collection of baseline or follow-up measures.

##### 2. Promotion of research prior to requests for participation

The research will be promoted to parents from all participating long day care services via a brochure disseminated a week prior to distribution of information and consent materials.

##### 3. Dissemination of materials to maximise parent engagement

The recruitment coordinator will arrange for recruitment packs (one per parent of each child aged three to five years) to be delivered to each participating service. Distribution of these packs to parents will occur via methods considered appropriate and most effective by the Authorised Supervisor. The research team will aim to hand recruitment packs directly to parents when they drop-off or pick-up their children from the long day care service. This will also enable parents to ask research staff questions about the research. Other distribution methods may include the service emailing parents or placing recruitment packs in children's pigeon holes, lockers or bags. The recruitment packs will be brightly coloured and include an information sheet, consent form and return envelope.

##### 4. Parent reminders

Two weeks after delivery of the recruitment packs, reminder letters will be disseminated via the same channels as described above. The letters will remind parents about the study and the opportunity to participate.

Parents will be asked to sign and return the consent form in the envelope provided to the service their child attends. Parents will have up to three weeks to return their consent form. The consent form includes items that ask for some demographic information about the parent and child, the usual number of days their child attends the service each week, and the after care physical activity and small screen recreation habits of their child on a usual week day. In order to identify any bias due to selective non-participation, all parents will be asked to complete the items on the consent form and return it regardless of whether they consent to study participation.

### Random allocation of long day care services

Services will be allocated to either the intervention or control condition using block randomization performed in a 1:1 ratio in randomly sequenced blocks of two, four or six by a computerized random number function in Microsoft Excel. Allocation of services will be undertaken by a statistician who will have no other involvement in the study, and will occur after all services have been recruited into the trial. As evidence suggests physical activity practices in long day care services differ according to the socio-economic status of the area in which the service is located [26], the random allocation of long day care services will be stratified by the socioeconomic characteristics (high/low) of the service locality. Long day care services with a postcode ranked in the top 50% of New South Wales, based on the Socio-Economic Indexes for Areas [27] will be defined as a 'high socio-economic area service' and those with a postcode ranked in the lower 50% will be defined as a 'low socio-economic area service'. Due to the difficulty in blinding services to their group allocation, this trial will be an 'open' trial. After services have consented to participate in the study a member of the research team not involved in recruitment or data collection will inform services of the group to which they were allocated.

### Intervention

#### Theoretical perspective

It is suggested that the effectiveness of interventions are maximised when an appropriate theoretical framework is utilised to guide intervention development [28]. The multi-level intervention, described below, has been designed using social ecological models of health behaviour change. Social ecological approaches acknowledge the multiple interrelated influences on health behaviours across social, cultural, and environmental domains [29,30]. The social ecological framework has been identified as a suitable conceptual model for the design of physical activity interventions [31] and has been applied when describing correlates of children's physical activity behaviours [6,32]. Furthermore, school-based interventions grounded in such social ecological theory have been found to be effective in increasing physical activity levels of children by altering instructional practices and the school environment [33]. Drawing on a social ecological framework, the intervention aims to influence children's physical activity behaviour through the manipulation of mediators across the social, physical and organisational environment of childcare services [12,34]. Specifically, the intervention will target staff instructional practices and interactions with children (social), service physical activity policy and programming (organisational) and the characteristics and equipment available within play space (physical environment).

#### Physical activity intervention

The intervention components are consistent with the recommendations of the draft Australian National Physical Activity guidelines for children [6] and the Australian National Healthy Eating and Physical Activity Guidelines for Early Childhood Services [21]. The intervention has been designed and will be overseen by an advisory group with representation from the Department of Community Services, the New South Wales Health Department, Authorised Supervisors from local services, health promotion practitioners, paediatric researchers and physical activity experts. The intervention will be delivered by staff of participating intervention group long day care services. Based on evidence from descriptive and available experimental research to increase child physical activity levels and reduce time spent being sedentary in childcare [11,21,35], the intervention will comprise of the following components:

*1. Delivering structured fundamental movement skill development activities *[9,18,35-40]:

Service staff will deliver daily structured fundamental movement skills (FMS) activities. Fundamental movement skills are the building blocks to more advanced movement skills and specific sport skills [41]. Structured activity is defined as those that are teacher initiated. Each session will include a warm up activity, age and developmentally appropriate teacher led games focusing on one or more FMS, and a cool down activity.

*2. Increasing the number of children's opportunities each day to participate in physical activity *[9]:

Service staff will increase the opportunities provided throughout the day for children to participate in physically active play. This will occur through service staff programming and opportunistically initiating movement based group activities such as dance and group games. This will also include modifying planned activities to incorporate active movement such as transitions between daily activities (such as moving inside to eat lunch or washing hands) and including movement within typically sedentary activities (such as table play e.g puzzles or play dough).

*3. Increasing staff role modelling of active play and delivery of instructional practices *[1,12,21,35]:

Staff will be supported to become active participants during all child initiated free play (role modelling) and provide verbal guidance (prompts to extend active play) and encouragement (positive statements about children's activity) to children to increase physical activity levels.

*4. Providing children with a physical activity promoting indoor and outdoor physical environment *[11,12,35,42-44]:

Services will increase the variety of activity promoting resources and toys available to children in indoor and outdoor areas. This will include varying arrangements of specific portable equipment to maximise child utilisation and interest. Services will also promote physically active play through displays, photos, books and posters within the service.

*5. Limiting children's small screen recreation and sedentary behaviours *[6,21]:

Whilst at the service, the amount of time children spend watching or using electronic media will be limited according to current aged based recommendations[6]. The time children spend in sedentary activities will be limited to periods of less than 30 minutes at a time (except when eating meals or sleeping).

#### Intervention implementation strategies

The research team will implement a number of strategies to engage services and facilitate their implementation of the physical activity intervention. The strategies to support intervention delivery are based on an organisational and practice change theoretical framework [45] and are supported empirically [46-50]. Specifically, the intervention implementation support strategies will include:

*1. Provision of staff training *[51,52]

All staff from intervention services will be invited to participate in a six-hour workshop to facilitate the implementation of the intervention. The workshop will introduce key physical activity intervention messages and concepts, include demonstrations of intervention activities and familiarisation with intervention resources. The training will support integration of physical activity across other learning areas linking to the service's existing curriculum, programs and activities [12,18,20]. The content of the workshop has been piloted with long day care services in the New England Region of New South Wales, Australia.

*2. Provision of resources and instructional materials *[36]

All services will receive a package of resources and instructional materials to sufficiently equip staff to implement the intervention. Specifically, the resources will include: an intervention manual providing a program rationale and background, current recommendations and best practice guidelines for physical activity in childcare services; policy template; instructional handbooks and DVD with age and developmentally appropriate physical activity games and play based activities to encourage the development of FMS; laminated activity cards to be used in the classroom with visual and written instructions for setting up and facilitating play based FMS activities; and lanyards to be worn by staff during outdoor play with pictures of each FMS including prompts to support teacher demonstration and cues for appropriate teaching. Services will also receive a planning resource in which to develop and record strategies for an individualised service action plan.

*3. Follow-up support *[50,53]

Authorised Supervisors will receive two 15 minute telephone support calls and a two hour service visit from intervention support staff to support the ongoing implementation of intervention components. The telephone support will be provided to Authorised Supervisors at approximately four and 15 weeks post provision of staff training. The service visit will occur approximately seven weeks post training. During the follow-up contacts, intervention support staff will assist Authorised Supervisor to set goals and develop an action plan regarding intervention delivery, review goals and service progress, reinforce service level changes and assist with problem solving. Authorised Supervisors will be asked to document goals, action plans and progress in a booklet provided. Additionally, during the service visits, intervention support staff will discuss any issues that service staff may be experiencing regarding the provision of intervention support.

*4. Performance monitoring, and feedback *[50,53]

Information collected during support contacts with the service will be used to monitor adoption of intervention components. Aggregated and non-identifiable summaries regarding implementation performance will be distributed to all services following the service visit and second phone contact via a project newsletter. The newsletter will reinforce the intervention components services are implementing well, highlight areas where some services may require improvement, and provide supportive information or case studies to facilitate intervention improvement. Performance feedback regarding individual service implementation will also be provided by program intervention staff during the follow-up service contacts.

*5. Use of relevant and credible opinion leaders *[46,51,52]

Support to services to deliver the intervention will be provided by two qualified early childhood teachers. The first represents a well known early childhood training organisation with extensive experience in the provision of training and support for services, particularly with regard to issues of child health. The second is a local practicing Authorised Supervisor, early childhood teacher and University Lecturer. Both intervention support staff members were selected on the advice of the Program Advisory Group as they are well known, influential and respected experts in the field of physical activity and early childhood, and would be perceived as both a credible and reliable source of information by Authorised Supervisors and service staff.

*6. Securing executive support and endorsement *[46]

The importance and benefits of implementing the physical activity intervention will be communicated to Authorised Supervisors and staff during telephone support calls, service visits and through the dissemination of regular project newsletters describing the implementation success of other services. Authorised Supervisors will be encouraged to demonstrate executive level support for the implementation and integration of the physical activity intervention into usual service practice through the endorsement and dissemination of a service level physical activity policy to staff and parents, and discussing service physical activity practices at staff meetings.

### Control group

Participating services randomised to the wait list control group will not receive any intervention support or materials during the study period. All control services will be offered staff training, resources and follow-up support after completion of all follow-up data collection.

### Data collection procedures

Research staff involved in data collection will be blind to group allocation and participating services will be asked not to disclose their group allocation to data collection staff during data collection. To assess the effectiveness of blinding, field data collection staff will be asked to guess the group to which they suspect the service was allocated following collection of trial outcome data.

#### Long day care service operational characteristics

To describe the operational characteristics of participating long day care services information will be collected from the Authorised Supervisor via telephone interview during the recruitment process.

#### Parent and child demographics and physical activity

Parents will be asked to complete items assessing basic demographic information about their child and their child's usual outside of care physical activity on the participant consent form at baseline. At follow-up, parents will again be asked to complete the items assessing child physical activity outside of care via a form which they will return to their childcare service. Self reported physical activity data will be used to assess any physical activity displacement as a result of the intervention.

#### Intervention implementation

Information on the implementation of the intervention by staff at each service will be collected via a staff survey, completion of the Environment and Policy Assessment Observation (EPAO) instrument and an audit of service documents.

The pen and paper staff survey will be distributed to all teaching staff at each participating service by the research team two weeks prior to baseline and follow-up collection of physical activity data. The survey contains items developed by the research team and takes approximately ten minutes to complete. Surveys will be coded to ensure answers remain confidential. Completed surveys will be posted back to the research coordinator or collected by field staff when they visit the service for data collection. The survey will measure the extent to which staff within each service implemented the intervention components as intended.

The physical activity component of the EPAO [54] will be used to assess intervention delivery during a one day field observation of staff practices, and environmental characteristics. The EPAO will be conducted in intervention and control services at baseline and follow-up on the day of field data collection assessing child physical activity. Two trained research staff will visit each service. The first staff member will act as the observer and record observations using the observation tool on one class of children aged three to five years at each service. Where a service has multiple classes, one class will be randomly selected to be the subject of observations. Observations will take place between 9 am to 3 pm, the core hours of service operation. The second staff member will assist with pedometer placement, playground measurement and general administration. The EPAO has been used in both descriptive and intervention studies [2,54] and has previously demonstrated high inter-observer agreement (87.3%) [54].

As part of the EPAO, one research staff member will a conduct brief ten minute interview with Authorised Supervisors where key physical activity documents including service policies and any physical activity curriculum will be viewed and audited. Data collection will be rescheduled in instances where weather conditions disrupt usual service routines and prevent children from using outdoor space (e.g during wet weather or temperature extremes).

#### Physical activity

Data will be collected from children attending each intervention and control service on a day of the week negotiated between the Authorised Supervisor and the research team. All children participating in the study will be asked to wear a pedometer (model Yamax SW200 and SW7000) on one week day over a six hour measurement period between 9 am and 3 pm. For each service data will be collected on the same day of the week at baseline and follow-up.

Pedometers are unobtrusive battery-operated instruments that are lightweight and about the size of a match-box. Pedometers measure vertical oscillations of body movement [55], and provide a total count of accumulated movements over the data collection time period [56]. Pedometers have been identified as a suitable tool for large-scale studies given their low cost and feasibility [56,57]. Additionally, pedometers have been demonstrated to be an accurate and reliable method of measuring physical activity levels in children [56,58] and preschool aged children [6,56,59]. Participant burden associated with wearing a pedometer is minimal [59], furthermore, it has been found that pre-school age children are comfortable with the contact required to collect data utilising pedometers [56].

The procedures for fitting participants with pedometers will follow protocols utilised in previous studies with young children [42,56,58]. Specifically, pedometers will be attached by trained research staff to the clothing of children above the right hip and in line with the right knee. If children wear dresses, loose pants or shorts, the pedometer will be attached to a small adjustable elastic belt worn by children at the waist. Pedometers will be set to zero at the beginning of the measurement period. Total step counts will be collected by research staff at the end of the measurement period. Pedometer data collection will also be rescheduled in instances where weather conditions disrupt usual service routines.

#### Intervention acceptability

Information on the acceptability of the intervention and intervention resources will be collected through the inclusion of items in the staff survey at follow-up for intervention services only.

### Measures

#### Long day care service operational characteristics

Operational information sought from the service will include the number of years in operation, the number of enrolled and attending children aged three, four and five years, and the number of primary contact teaching staff.

#### Parent and child demographics and physical activity

Parents will be asked to report child age, Aboriginal and/or Torres Strait Islander status, gender, postcode of residence and parental education level on the participant consent form. Parents will also be asked about the usual number of days their child spends at long day care each week and the usual amount of time their child spends being physically active and participating in small screen recreation during weekdays outside of care hours. Items assessing demographic and time spent in physical activity and small screen recreation outside of care were based on those used in other population based surveys of pre-school age Australian children [60].

#### Intervention implementation

Triangulation will be used to assess the extent to which services implemented the intervention as intended. First, data from the staff survey will assess how often staff report delivering structured fundamental movement skill activities for three to five year olds; the inclusion of warm ups, cool downs and skill specific feedback in FMS activities and the usual amount of time that structured FMS activities run for. The survey will also assess the frequency with which service staff report delivering verbal prompts and participating in children's active play; the number of occasions per day that the majority of three to five year old children are sedentary for over 30 minutes at a time (excluding meal and nap times); and how often and how long three to five year old children participate in small screen recreation activities.

Second, the EPAO field study will provide observational information on key physical activity intervention components occurring at the service on the day of data collection. This will include the number of occasions and total minutes of outdoor play, teacher led physical activities and structured fundamental movement skills activities during the six hour observation period. The number of times during the observation period that staff deliver prompts to increase activity and make positive statements to encourage activity, the number of times staff join in children's active play, and the total minutes children spend in sedentary activities or small screen recreation. The observation will include identifying the presence of portable and fixed play equipment in indoor and outdoor areas, a description of the space available for indoor and outdoor play (limited room for active play, obstructed by furniture or equipment), and a checklist of features of the outdoor play space such as playground surfaces and markings, vegetation and the presence of physical activity displays, books and posters.

Third, data collected as part of the EPAO Authorised Supervisor interview and service audit will be used to assess the presence of a physical activity policy, support within the policy for limiting small screen recreation time, integrating physical activity into the curricula and the provision of daily fundamental movement skills activities.

#### Physical activity

The primary trial outcome is child physical activity, operationally defined as step counts per minute [35,42,56] as measured by pedometers over the six hour operational period of services, from 9 am to 3 pm.

#### Intervention acceptability

At follow-up, the intervention service staff survey will include items assessing the use, acceptability and satisfaction with the intervention training and support provided to staff and services as part of the intervention. The items will require staff to respond to a series of statements on a four point Likert scale ranging from 'strongly agree' to 'strongly disagree'. Acceptability items were developed by the research team based on previous assessments of staff acceptability in delivering health promotion programs [61].

### Sample size and power calculations

Assuming a step count per minute of 17 among children attending control services and an intra-class correlation of 0.1 [62] a sample size of approximately 280 children (140 per group) attending 20 services at the 6 month follow-up will be sufficient to detect a difference between intervention and control groups of 4 steps per minute with 80% power at the 0.05 significance level. Assuming that long day care services care for 30 children aged three to five years per day on average, a study participation rate of 65% will be required to obtain the desired sample given a 20% attrition rate at the follow-up assessments.

### Analysis

All statistical analyses will be performed with SAS (version 9.2 or later) statistical software. All statistical tests will be two tailed with an alpha value of 0.5. Descriptive statistics will be performed to describe the demographic and service characteristics of intervention and control groups at baseline. Similarly measures of intervention implementation will be described using descriptive statistics.

The effectiveness of the intervention on child physical activity will be assessed utilising an intention to treat approach. An intention to treat analysis includes all participants in the analysis based on the groups to which they were allocated, without excluding data based on missing outcomes or non-adherence [22]. Specifically, linear mixed models will be used to examine between group differences on the primary trial outcome. Such analyses account for the correlation between pre and post measures and adjust for clustering. Any differences in the characteristics of participants at baseline will be adjusted for in the final linear model.

To ensure the results are robust, a sensitivity analysis will be performed whereby participants' observations at baseline will be used as a substitute for any subsequent missing data. A per-protocol analysis will also be conducted with participants from services which have sufficiently implemented the intervention.

Acceptability of the intervention among staff of services will be assessed by collapsing Likert scale categories and reporting the percentage of staff who responded 'strongly agree' or 'agree' to each acceptability item.

## Discussion

There is a clear need for intervention studies to extend research regarding the effectiveness of interventions to increase physical activity behaviours of young children attending childcare [17]. This trial aims to advance the currently limited experimental evidence in this field and will contribute important information regarding the effectiveness, feasibility and acceptability of comprehensive service based strategies to address physical activity at childcare. Strengths of this study include the trials randomised design, the use of theory and multi-disciplinary input into the intervention design, the implementation of the intervention by usual service staff, and the use of an objective measure of physical activity.

## Conclusion

This manuscript provides a description of the implementation of a cluster randomised controlled trial of a multi-component intervention aimed at increasing physical activity levels of preschool aged children attending long day care services. The study is one of a handful of randomised trials of such interventions internationally and will contribute greatly to the evidence regarding the effectiveness of strategies in this setting.

## Competing interests

The authors declare that they have no competing interests.

## Authors' contributions

First author MFi led the development of this manuscript. Authors LW, PM, MFi and JW conceived the intervention concept. Authors LW, PM, JW and MFr secured grant funding from Hunter Medical Research Institute. Author RW contributed to the development of the recruitment protocol. All authors contributed to the research design and trial methodology and contributed to, read and approved the final version of this manuscript.

## Pre-publication history

The pre-publication history for this paper can be accessed here:

http://www.biomedcentral.com/1471-2458/10/534/prepub

## References

[B1] Timmons BW Naylor PJ Pfeiffer KA Physical activity for preschool children--how much and how? Canadian Journal of Public Health Revue Canadienne de Sante Publique 2007 98 Suppl 2 S122 134 18213943

[B2] McWilliams C Ball SC Benjamin SE Hales D Vaughn A Ward DS Best-Practice Guidelines for Physical Activity at Child Care Pediatrics 2009 124 6 1650 1659 10.1542/peds.2009-0952 19917582

[B3] Oliver M Schofield GM Kolt GS Physical Activity in Preschoolers: Understanding Prevalence and Measurement Issues Sports Medicine 2007 37 1045 1070 10.2165/00007256-200737120-00004 18027993

[B4] Reilly JJ Penpraze V Hislop J Davies G Grant S Paton JY Objective measurement of physical activity and sedentary behaviour: review with new data Archives of Disease in Childhood 2008 93 7 614 619 10.1136/adc.2007.133272 18305072

[B5] Ward DS Physical activity in young children: the role of child care Medicine & Science in Sports & Exercise 2010 42 3 499 501 10.1249/MSS.0b013e3181ce9f85 20068500

[B6] Okely AD Salmon J Trost SG Hinkley T Discussion paper for the development of physical activity recommendations for children under five years 2008 Canberra: Australian Department of Health and Ageing

[B7] Taylor RW Murdoch L Carter P Gerrard DF Williams SM Taylor BJ Longitudinal Study of Physical Activity and Inactivity in Preschoolers: The FLAME Study Medicine & Science in Sports & Exercise 2009 41 1 96 102 10.1249/MSS.0b013e3181849d81 19092702

[B8] Okely AD Trost SG Steele JR Cliff DP Mickle K Adherence to physical activity and electronic media guidelines in Australian pre-school children Journal of Paediatrics and Child Health 2009 45 1-2 5 8 10.1111/j.1440-1754.2008.01445.x 19208059

[B9] Bower JK Hales DP Tate DF Rubin DA Benjamin SE Ward DS The Childcare Environment and Children's Physical Activity American Journal of Preventive Medicine 2008 34 1 23 29 10.1016/j.amepre.2007.09.022 18083447

[B10] Finn K Johannsen N Specker B Factors associated with physical activity in preschool children The Journal of Pediatrics 2002 140 1 81 85 10.1067/mpd.2002.120693 11815768

[B11] Dowda M Brown WH McIver KL Pfeiffer KA O'Neill JR Addy CL Pate RR Dowda M Brown WH McIver KL Policies and characteristics of the preschool environment and physical activity of young children Pediatrics 2009 123 2 e261 266 10.1542/peds.2008-2498 19171578 PMC2632768

[B12] Trost SG Ward DS Senso M Trost SG Ward DS Senso M Effects of child care policy and environment on physical activity Medicine & Science in Sports & Exercise 2010 42 3 520 525 10.1249/MSS.0b013e3181cea3ef 20068496

[B13] Story M Kaphingst KM French S The role of child care settings in obesity prevention Future of Children 2006 16 1 143 168 10.1353/foc.2006.0010 16532662

[B14] Australian Bureau of Statistics Childhood Education and Care. 4402.0 Canberra 2008 (Reissue)

[B15] Cashmore A Jones S Growing Up Active: A Study Into Physical Activity in Long Day Care Centers Journal of Research in Childhood Education 2008 23 2 179 10.1080/02568540809594654

[B16] Pagnini D Wilkenfeld R King L Booth M Booth S The Weight of Opinion: The early childhood sector's perceptions about childhood overweight and obesity 2006 Sydney: NSW Centre for Overweight and Obesity 10.1071/he07149 17663651

[B17] Trost SG Ward DS Senso M Effects of child care policy and environment on physical activity Medicine & Science in Sports & Exercise 2010 42 3 520 525 10.1249/MSS.0b013e3181cea3ef 20068496

[B18] Ward DS Vaughn A McWilliams C Hales D Interventions for increasing physical activity at child care Medicine & Science in Sports & Exercise 2010 42 3 526 534 10.1249/MSS.0b013e3181cea406 20068495

[B19] Alhassan S Sirard JR Robinson TN The effects of increasing outdoor play time on physical activity in Latino preschool children International Journal of Pediatric Obesity 2007 2 3 153 158 10.1080/17477160701520108 17852547

[B20] Trost SG Fees B Dzewaltowski D Feasibility and Efficacy of a "Move and Learn" Physical Activity Curriculum in Preschool Children Journal of Physical Activity and Health 2008 5 1 88 103 18209256 10.1123/jpah.5.1.88

[B21] Australian Government Department of Health and Ageing Get Up and Grow: Healthy Eating and Physical Activity for Early Childhood:Director/Coordinator Book Canberra 2009

[B22] Moher D Schulz KF Altman DG Group C CONSORT 2010 statement: updated guidelines for reporting parallel group randomised trials PLoS Medicine/Public Library of Science 2010 7 3 e1000251 10.1371/journal.pmed.1000251 PMC2844794 20352064

[B23] New South Wales Department of Health Population Health Division:The Health of the People of New South Wales - Report of the Chief Health Officer Sydney 2006

[B24] Australian Bureau of Statistics 2006 Census of Population Health and Housing Canberra 2007

[B25] Wolfenden L Kypri K Freund M Hodder R Obtaining active parental consent for school-based research: a guide for researchers Australian & New Zealand Journal of Public Health 2009 33 3 270 275 10.1111/j.1753-6405.2009.00387.x 19630848

[B26] Wolfenden L Neve M Farrell L Lecanthelinais C Bell A Milat A Wiggers J Physical activity policies and practices of chilcare centres in Australia Journal of Paediatrics and Child Health 2010 in press 10.1111/j.1440-1754.2010.01738.x 20500433

[B27] Australian Bureau of Statistics An introduction to SocioEconomic Indexes for Areas (SEIFA) Canberra 2006

[B28] Stokols D Allen J Bellingham RL The social ecology of health promotion: implications for research and practice American Journal of Health Promotion 1996 10 4 247 251 10159704 10.4278/0890-1171-10.4.247

[B29] McLeroy KR Bibeau D Steckler A Glanz K An ecological perspective on health promotion programs Health Education Quarterly 1988 15 4 351 377 3068205 10.1177/109019818801500401

[B30] Stokols D Establishing and maintaining healthy environments. Toward a social ecology of health promotion American Psychologist 1992 47 1 6 22 10.1037/0003-066X.47.1.6 1539925

[B31] King AC Stokols D Talen E Brassington GS Killingsworth R Theoretical approaches to the promotion of physical activity: forging a transdisciplinary paradigm American Journal of Preventive Medicine 2002 23 2 Suppl 15 25 10.1016/S0749-3797(02)00470-1 12133734

[B32] Sallis JF Nader PR Broyles SL Berry CC Elder JP McKenzie TL Nelson JA Correlates of Physical Activity at Home in Mexican-American and Anglo-American Preschool Children Health Psychology 1993 12 5 390 398 10.1037/0278-6133.12.5.390 8223363

[B33] Pate RR Ward DS Saunders RP Felton G Promotion of Physical Activity Among High-School Girls: A Randomized Controlled Trial American Journal of Public Health 2005 95 9 1582 10.2105/AJPH.2004.045807 16118370 PMC1449401

[B34] Stokols D Translating social ecological theory into guidelines for community health promotion American Journal of Health Promotion 1996 10 4 282 298 10159709 10.4278/0890-1171-10.4.282

[B35] Cardon G Labarque V Smits D Bourdeaudhuij ID Promoting physical activity at the pre-school playground: The effects of providing markings and play equipment Preventive Medicine 2009 48 4 335 340 10.1016/j.ypmed.2009.02.013 19236894

[B36] Hardy LL King L Farrell L Macniven R Howlett S Fundamental movement skills among Australian preschool children Journal of Science and Medicine in Sport 2010 13 5 503 508 10.1016/j.jsams.2009.05.010 19850520

[B37] Barnett LM van Beurden E Morgan PJ Brooks LO Beard JR Childhood Motor Skill Proficiency as a Predictor of Adolescent Physical Activity Journal of Adolescent Health 2009 44 3 252 259 10.1016/j.jadohealth.2008.07.004 19237111

[B38] Cliff DP Okely AD Smith LM Kim M Relationships Between Fundamental Movement Skills and Objectively Measured Physical Activity in Preschool Children Pediatric Exercise Science 2009 21 4 436 449 20128363 10.1123/pes.21.4.436

[B39] Williams HG Pfeiffer KA O'Neill JR Dowda M McIver KL Brown WH Pate RR Motor Skill Performance and Physical Activity in Preschool Children Obesity 2008 16 6 1421 1426 10.1038/oby.2008.214 18388895

[B40] Lubans DR Morgan PJ Cliff DP Barnett LM Okely AD Fundamental movement skills in children and adolescents: Review of associated health benefits Sports Medicine in press (Accepted 20 March, 2010) 10.2165/11536850-000000000-00000 21058749

[B41] Goodway JD Branta CF Influence of a motor skill intervention on fundamental motor skill development of disadvantaged preschool children Research Quarterly for Exercise and Sport 2003 74 1 36 12659474 10.1080/02701367.2003.10609062

[B42] Boldemann C Blennow M Dal H Mårtensson F Raustorp A Yuen K Wester U Impact of preschool environment upon children's physical activity and sun exposure Preventive Medicine 2006 42 4 301 308 10.1016/j.ypmed.2005.12.006 16448688

[B43] Timmons BW Naylor PJ Pfeiffer KA Physical activity for preschool children - how much and how? Applied Physiology, Nutrition & Metabolism 2007 32 S122 S134 10.1139/H07-166 19377537

[B44] Dhingra R Manhas S Raina A Play pattern in preschool setting Journal of Human Ecology 2005 18 1 21 25

[B45] Moulding NT Silagy CA Weller DP A framework for effective management of change in clinical practice: dissemination and implementation of clinical practice guidelines Quality in Health Care 1999 8 3 177 183 10.1136/qshc.8.3.177 10847875 PMC2483658

[B46] Oxman AD Thomson MA Davis DA Haynes RB No magic bullets: a systematic review of 102 trials of interventions to improve professional practice CMAJ Canadian Medical Association Journal 1995 153 10 1423 1431 PMC1487455 7585368

[B47] Hulscher MWM van der Weijden T Grol R Interventions to implement prevention in primary care (Cochrane Review) Cochrane Database of Systematic Reviews 2003 1 CD000362 10.1002/14651858.CD000362 11279688

[B48] Green L M K Health Promotion Planning and Evaluation: An Educational and Environmental Approach 1991 Mountain View, Ca: Mayfield Publishing

[B49] Bero LA Grilli R Grimshaw JM Harvey E Oxman AD Thomson MA Closing the gap between research and practice: an overview of systematic reviews of interventions to promote the implementation of research findings. The Cochrane Effective Practice and Organization of Care Review Group BMJ 1998 317 7156 465 468 9703533 10.1136/bmj.317.7156.465 PMC1113716

[B50] Soumerai SB Avorn J Principles of educational outreach ('academic detailing') to improve clinical decision making JAMA 1990 263 4 549 556 10.1001/jama.263.4.549 2104640

[B51] Rosenthal MS Crowley AA Curry L Promoting child development and behavioral health: family child care providers' perspectives Journal of Pediatric Health Care 2009 23 5 289 297 10.1016/j.pedhc.2008.08.001 19720263

[B52] Fees B Trost S Bopp M Dzewaltowski DA Physical Activity Programming in Family Child Care Homes: Providers' Perceptions of Practices and Barriers Journal of Nutrition Education and Behavior 2009 41 4 268 273 10.1016/j.jneb.2008.01.013 19508932

[B53] Abraham C Michie S Abraham C Michie S A taxonomy of behavior change techniques used in interventions Health Psychology 2008 27 3 379 387 10.1037/0278-6133.27.3.379 18624603

[B54] Ward DE Hales DP Haverly KM Marks JP Benjamin SP Ball SMR Trost SP An Instrument to Assess the Obesogenic Environment of Child Care Centers American Journal of Health Behavior 2008 32 4 380 18092898 10.5555/ajhb.2008.32.4.380

[B55] Louie L Chan L The Use of Pedometry to Evaluate the Physical Activity Levels among Preschool Children in Hong Kong Early Child Development & Care 2003 173 1 97 107

[B56] McKee DP Boreham CAG Murphy MH Nevill AM Validation of the Digiwalker(tm) Pedometer for Measuring Physical Activity in Young Children Pediatric Exercise Science 2005 17 4 345

[B57] Tudor-Locke C Williams JE Reis JP Pluto D Utility of pedometers for assessing physical activity: convergent validity Sports Medicine 2002 32 12 795 808 10.2165/00007256-200232120-00004 12238942

[B58] Cardon G De Bourdeaudhuij I Comparison of Pedometer and Accelerometer Measures of Physical Activity in Preschool Children Pediatric Exercise Science 2007 19 2 205 214 17603143 10.1123/pes.19.2.205

[B59] Pate RR O'Neill JR Mitchell J Measurement of Physical Activity in Preschool Children Medicine & Science in Sports & Exercise 2010 42 3 508 12 10.1249/MSS.0b013e3181cea116 20068498

[B60] New South Wales Department of Health (HOIST) New South Wales Population Health Survey 2009 118 Sydney: Centre for Epidemiology and Research

[B61] Wolfenden L Wiggers J Campbell E Knight J Kerridge R Moore K Spigelman A Harrison M Wolfenden L Wiggers J Feasibility, acceptability, and cost of referring surgical patients for postdischarge cessation support from a quitline Nicotine & Tobacco Research 2008 10 6 1105 1108 10.1080/14622200802097472 18584474

[B62] Reilly JJ Kelly L Montgomery C Williamson A Fisher A McColl JH Lo Conte R Paton JY Grant S Physical activity to prevent obesity in young children: cluster randomised controlled trial BMJ 2006 333 7577 1041 10.1136/bmj.38979.623773.55 17028105 PMC1647320

